# Progressive grey matter atrophy in adolescents with major depressive disorder revealed by causal structural covariance network

**DOI:** 10.1192/j.eurpsy.2024.413

**Published:** 2024-08-27

**Authors:** J. Chen, X. Jin, J. Gao, Y. Zhang, C. Bai, F. Xu, Y. Yao, D. Yu, Y. Yang, W. Zhang, X. Zhu, K. Wang

**Affiliations:** ^1^School of Psychology, Shandong Normal University; ^2^Department of Radiology, Qilu Hospital of Shandong University; ^3^Childhood Psychiatry Unit, Shandong Mental Health Center, Jinan, China; ^4^School of Health and Wellbeing, University of Glasgow, Glasgow, United Kingdom

## Abstract

**Introduction:**

Adolescence is a period marked by highest vulnerability to the onset of depression, with profound implications for adult health. Neuroimaging studies have revealed considerable atrophy in brain structure in these patients with depression. Of particular importance are regions responsible for cognitive control, reward, and self-referential processing. However, the causal structural networks underpinning brain region atrophies in adolescents with depression remain unclear.

**Objectives:**

This study aimed to investigate the temporal course and causal relationships of gray matter atrophy within the brains of adolescents with depression.

**Methods:**

We analyzed T1-weighted structural images using voxel-based morphometry in first-episode adolescent patients with depression (n=80, 22 males; age = 15.57±1.78) and age, gender matched healthy controls (n=82, 25 males; age = 16.11±2.76) to identify the disease stage-specific gray matter abnormalities. Then, with granger causality analysis, we arranged the patients’ illness duration chronologically to construct the causal structural covariance networks that investigated the causal relationships of those atypical structures.

**Results:**

Compared to controls, smaller volumes in ventral medial prefrontal cortex (vmPFC), dorsal anterior cingulate cortex (dACC), middle cingulate cortex (MCC) and insula areas were identified in patients with less than 1 year illness duration, and further progressed to the subgenual ACC, regions of default, frontoparietal networks in longer duration. Causal network results revealed that dACC, vmPFC, MCC and insula were prominent nodes projecting exerted positive causal effects to regions of the default mode and frontoparietal networks. The dACC, vmPFC and insula also had positive projections to the reward network, which included mainly the thalamus, caudate and putamen, while MCC also exerted a positive causal effect on the insula and thalamus.

**Conclusions:**

These findings revealed the progression of structural atrophy in adolescent patients with depression and demonstrated the causal relationships between regions involving cognitive control, reward and self-referential processes.

**Disclosure of Interest:**

None Declared

